# 异基因造血干细胞移植后女性血液系统疾病患者生育力情况的回顾性研究

**DOI:** 10.3760/cma.j.cn121090-20250630-00307

**Published:** 2025-12

**Authors:** 佳怡 黄, 吴思弦 黄, 博宣 蒋, 傅文萱 丁, 苏宁 陈, 文治 蔡

**Affiliations:** 苏州大学附属第一医院血液科，国家血液系统疾病临床医学研究中心，江苏省血液研究所，苏州 215006 Department of Hematology, the First Affiliated Hospital of Soochow University, National Clinical Research Center for Hematologic Diseases, Jiangsu Institute of Hematology, Suzhou 215006, China

**Keywords:** 女性, 血液病, 异基因造血干细胞移植, 生育力保护和保存, Femininity, Hematologic diseases, Allogeneic hematopoietic stem cell transplantation, Fertility preservation and conservation

## Abstract

**目的:**

探讨异基因造血干细胞移植（allo-HSCT）对女性血液系统疾病患者生育力的影响以及生育力保护和保存措施的可行性。

**方法:**

回顾性分析2012年5月至2024年1月苏州大学附属第一医院血液科263例移植前无生育史的女性血液系统疾病患者的疾病类型、移植前治疗、预处理方案、生育力保护措施等与患者移植后月经恢复及生育情况的相关性。采用卡方检验进行组间比较，Logistic回归模型探讨影响月经恢复的独立危险因素。

**结果:**

263例患者中，228例（86.7％）移植后继发闭经，35例（13.3％）移植后自然恢复月经。组间分析结果显示，预处理方案（*χ*²＝4.84，*P*＝0.028）与移植前生育力保护措施（*χ*²＝52.18，*P*<0.001）是月经恢复的影响因素。多因素分析显示，移植前使用促性腺激素释放激素激动剂（GnRH-a）是月经恢复的独立影响因素（*OR*＝0.06，95％ *CI*：0.03～0.15，*P*<0.001）。137例接受激素替代治疗（HRT）的患者中，134例（97.8％）恢复月经。组间比较显示，用药后月经恢复状况与移植年龄（*H*＝25.36，*P*<0.001）、预处理方案（*χ*²＝10.89，*P*＝0.004）、移植类型（*χ*²＝15.24，*P*＝0.003）、移植前使用GnRH-a（*χ*²＝65.18，*P*<0.001）及烷化剂（*χ*²＝6.35，*P*＝0.042）相关。中位随访83.80（59.13，108.37）个月，5例成功生育，其中1例自然妊娠，4例通过卵母细胞冷冻技术获得后代。

**结论:**

allo-HSCT对女性患者的生育力影响显著，移植前使用GnRH-a、卵母细胞冷冻，移植后行HRT等是年轻女性患者生育力保护和保存的有效手段。

## Abstract (en)

**Objective:**

This study aimed to investigate the impact of allogeneic hematopoietic stem cell transplantation（allo-HSCT）on fertility in female patients with hematological diseases as well as the feasibility of fertility preservation and conservation measures.

**Methods:**

This study retrospectively analyzed 263 nulliparous female patients undergoing allo-HSCT in the Department of Hematology at The First Affiliated Hospital of Soochow University from May 2012 to January 2024. Correlations between disease type, pretransplant therapies, conditioning regimens, fertility preservation measures, and posttransplant menstrual recovery/fertility outcomes were assessed. Chi-square tests were used for intergroup comparisons. Logistic regression identified independent risk factors for secondary amenorrhea.

**Results:**

Among the 263 patients, 228（86.7％）developed secondary amenorrhea after transplantation, whereas 35（13.3％）experienced natural recovery of menstruation. Intergroup analysis identified the conditioning regimen（*χ*^2^＝4.84, *P*＝0.028）and fertility protection measures before transplantation（*χ*^2^＝52.18, *P*<0.001）as influencing factors for menstrual recovery. Multivariate analysis revealed the use of GnRH-agonist（GnRH-a）before transplantation as an independent influencing factor for menstrual recovery（*OR*＝0.06, 95％ *CI*: 0.03–0.15, *P*<0.001）. Among 137 patients who received hormone replacement therapy（HRT）, 134（97.8％）resumed menstruation. Intergroup comparisons revealed that posttreatment menstrual recovery status was significantly associated with age at transplantation（*H*＝25.36, *P*<0.001）, conditioning regimen（*χ*^2^＝10.89, *P*＝0.004）, transplant type（*χ*^2^＝15.24, *P*＝0.003）, pretransplant use of GnRH-a（*χ*^2^＝65.18, *P*<0.001）, and alkylating agents（*χ*^2^＝6.35, *P*＝0.042）. With a median follow-up of 83.80（59.13, 108.37）months, 5 patients successfully gave birth, comprising 1 case of natural conception and 4 cases of achieving offspring through oocyte cryopreservation technology.

**Conclusion:**

Allo-HSCT significantly impacts fertility in female patients. Pretransplant GnRH-a, oocyte cryopreservation, and posttransplant HRT are effective methods for protecting and preserving fertility among young female patients.

女性血液系统疾病种类繁多，其中急性白血病（AL）、骨髓增生异常综合征（MDS）、骨髓增殖性肿瘤（MPN）、再生障碍性贫血（AA）、非霍奇金淋巴瘤（NHL）是异基因造血干细胞移植（allo-HSCT）的主要适应证。近年来，随着移植技术改进、移植物抗宿主病（GVHD）预防及管理策略的优化，全球移植例数逐年上升，移植后生存时间逐步延长。与此同时，越来越多育龄期女性患者在接受治疗后需要面对移植前强化化疗、移植预处理方案中烷化剂或放疗等诱导的卵巢功能衰竭问题。

治疗相关的卵巢早衰（POI）在女性移植患者中发生率可高达70％以上。AL等恶性血液病可直接浸润卵巢、破坏卵巢组织，抗白血病免疫系统激活导致的炎症因子释放和代谢异常也会对卵巢功能造成一定的损害[1]。治疗过程中的烷化剂，尤其是环磷酰胺，通过烷化DNA导致DNA交联和断裂，从而诱导卵泡凋亡和颗粒细胞损伤[2]，其对卵巢的毒性作用具有剂量和年龄依赖性[3]–[4]。全身照射（TBI）和局部放疗都会对卵巢造成直接损伤，包括卵泡耗竭和卵巢纤维化等[3]。POI通常表现为持续闭经、卵巢储备显著下降、内分泌失衡和生育能力丧失[5]–[6]。长期雌激素缺乏会导致女性患者骨密度降低、心血管风险增加，而生育力丧失则常伴随焦虑、抑郁及躯体化症状的加剧，严重影响其身心健康。

为此，本团队开展接受allo-HSCT治疗的女性血液系统疾病患者的系统性回顾性研究，分析移植患者卵巢功能和生育能力恢复的影响因素，探讨如何进一步将女性患者生育力保护和保存纳入到allo-HSCT的治疗过程，建立有效的生育力评估和保护策略，进而提升移植整体疗效。

## 病例与方法

一、病例

本研究为回顾性研究，研究对象纳入标准：①年龄>10岁且≤30岁女性；②诊断为血液系统疾病，包括急性髓系白血病、急性淋巴细胞白血病、NHL、AA、MDS、MPN；③接受allo-HSCT；④移植前无生育史。排除标准：①确认死亡患者；②失访患者。研究共纳入2012年5月至2024年1月在苏州大学附属第一医院接受allo-HSCT的263例女性血液系统疾病患者。该研究通过苏州大学附属第一医院医学伦理委员会批准，批件号为（2025）伦审批第537号，豁免患者签署知情同意书。

二、预处理治疗

清髓性预处理方案（MAC）：①以白消安（Bu）或环磷酰胺（Cy）为基础的预处理方案：司莫司汀250 mg/m^2^［移植前10天（−10 d）］，阿糖胞苷2 g/m^2^，每12小时1次（−9～−8 d），Bu 4 mg·kg^−1^·d^−1^（−7～−5 d），Cy 1.8 g·m^−2^·d^−1^（−4～−3 d）以及兔抗人胸腺细胞免疫球蛋白（rATG）2.5 mg·kg^−1^·d^−1^（−5～−2 d）[7]。②TBI/Cy方案：司莫司汀250 mg/m^2^（−8 d），羟基脲40 mg/kg，每12小时1次（−8 d），TBI 8 Gy（−7 d），阿糖胞苷2 g·m^−2^·d^−1^（−6～−5 d），Cy 60 mg·kg^−1^·d^−1^（−4～−3 d）[8]。

非清髓性预处理方案（NMAC）：①AA患者的Bu/Cy方案：具体为Bu 3.2 mg·kg^−1^·d^−1^（−7～−6 d），Cy 50 mg·kg^−1^·d^−1^（−5～−2 d），联合应用rATG（2.5 mg·kg^−1^·d^−1^）或抗淋巴细胞免疫球蛋白（20 mg·kg^−1^·d^−1^）（−5～−2 d）[9]。②FC+rATG方案：具体为氟达拉滨30 mg·m^−2^·d^−1^（−6～−2 d），Cy 30 mg·kg^−1^·d^−1^（−3～−2 d）及rATG 1.5 mg·kg^−1^·d^−1^（−5～−2 d）[10]。干细胞回输、GVHD预防、移植后疾病监测与本中心其他研究一致[7]–[9]。

三、生育力保护和保存方法

本研究从卵巢的生殖与内分泌功能角度，对行allo-HSCT女性患者的生育力状况进行综合评估。评估主要依据以下指标：①临床结局，即移植后能否出现自发性月经。②血清学生物标志物，以抗米勒管激素（AMH）水平作为评估卵巢储备功能的核心直接指标，并以促卵泡生成素（FSH）水平作为辅助判断依据。主要的生育力保护和保存方法包括：①促性腺激素释放激素激动剂（GnRH-a）：移植前预处理第1天皮下注射戈舍瑞林3.6 mg。②激素替代治疗（HRT）：以28 d为1个周期给药，口服雌二醇2 mg/d或结合雌激素0.625 mg/d；同时，在每个周期的第15至28天加用地屈孕酮，10 mg/d。③卵母细胞冷冻保存：患者在控制性超促排卵（COH）后超声引导下经阴道穿刺取卵和玻璃化冷冻。

四、指标分析与随访

回顾性分析患者的年龄、原发疾病种类及移植前放化疗方案、造血干细胞移植预处理方案、移植类型、卵巢储备功能、子宫状态评估、移植后月经情况、激素水平与妇科用药情况，相关数据主要通过移植后规律的门诊复查及电话随访获取。截止随访日期为2025年5月24日，中位随访时间为83.80（59.13，108.37）个月。

五、统计学处理

采用R Studio 4.5.1软件进行统计分析。连续变量以*M*（*Q*_1_，*Q*_3_）表示，分类变量采用频数（百分比）进行汇总，组间比较采用以下方法：分类变量：卡方检验；连续变量：Wilcoxon秩和检验，多因素分析采用Logistic回归模型探讨影响月经恢复及用药后月经恢复情况的因素。显著性水平定义为双侧*P*值小于0.05。*P*<0.05为差异具有统计学意义。

## 结果

一、基线特征

263例移植前未育的女性患者中位年龄21（16，25）岁，其中10～19岁110例、20～29岁153例；AA 37例、AL 194例、MDS/MPN 21例、NHL 11例。移植预处理方案方面，224例（85.2％）患者接受MAC，39例（14.8％）患者接受NMAC（包括37例AA、1例MDS/MPN和1例NHL）。供体来源方面，35例患者接受同胞全相合移植，202例接受亲缘半相合移植，26例患者接受无关全相合移植。其余基线特征信息详见表1。

**表1:** 263例接受异基因造血干细胞移植的女性血液系统疾病患者的基线资料［例（％）］

特征	总体（263例）	移植后继发闭经组（228例）	移植后自然恢复月经组（35例）	统计量	*P*值
移植年龄［岁，*M*（*Q*_1_，*Q*_3_）］	21（16，25）	21（16，25）	19（15，25）	*Z*＝−0.78	0.437
移植前生育力保护措施				*χ*^2^＝52.18	<0.001
卵母细胞冷冻	5（1.9）	3（1.3）	2（5.7）		
GnRH-a	51（19.4）	28（12.3）	23（65.7）		
未行生育力保护	207（78.7）	197（86.4）	10（28.6）		
疾病种类				*χ*^2^＝7.33	0.064
AA	37（14.1）	27（11.8）	10（28.6）		
AL	194（73.8）	173（75.9）	21（60.0）		
MDS/MPN	21（8.0）	18（7.9）	3（8.6）		
NHL	11（4.2）	10（4.4）	1（2.9）		
移植前月经情况				*χ*^2^＝2.31	0.511
青春期前状态	11（4.2）	8（3.5）	3（8.6）		
闭经	20（7.6）	18（7.9）	2（5.7）		
紊乱	21（8.0）	19（8.3）	2（5.7）		
正常	211（80.2）	183（80.3）	28（80.0）		
移植前化疗疗程［个，*M*（*Q*_1_，*Q*_3_）］	2（2，3）	3（2，3）	2（0，3）	*Z*＝−1.62	0.105
移植前使用烷化剂	106（40.3）	95（41.7）	11（31.4）	*χ*^2^＝0.93	0.335
预处理方案				*χ*^2^＝4.84	0.028
MAC	224（85.2）	199（87.3）	25（71.4）		
NMAC	39（14.8）	29（12.7）	10（28.6）		
移植类型				*χ*^2^＝5.81	0.055
同胞全相合	35（13.3）	32（14.0）	3（8.6）		
单倍型	202（76.8）	170（74.6）	32（91.4）		
无关全相合	26（9.9）	26（11.4）	0（0）		
粒系重建时间［d，*M*（*Q*_1_，*Q*_3_）］	11（11，12）	11（11，12）	11（11，12）	*Z*＝−0.44	0.660
GVHD	189（71.9）	163（71.5）	26（74.3）	*χ*^2^＝0.02	0.888
移植后性激素水平^a^［*M*（*Q*_1_，*Q*_3_）］					
黄体生成素（IU/L）	37.56（10.65，55.18）	36.43（10.90，53.65）	49.48（8.32，59.31）	*Z*＝−0.44	0.658
泌乳素（ng/ml）	12.21（8.94，18.85）	12.32（8.95，18.85）	9.80（8.23，17.88）	*Z*＝−0.61	0.545
卵泡刺激素（IU/L）	74.77（10.12，108.53）	69.13（10.12，107.07）	97.70（24.40，121.75）	*Z*＝−0.72	0.473
睾酮（ng/dl）	0.24（0.12，0.40）	0.23（0.12，0.41）	0.28（0.21，0.33）	*Z*＝−0.31	0.758
雌二醇（pg/ml）	14.00（12.00，25.30）	14.00（12.00，24.80）	13.50（11.75，34.50）	*Z*＝−0.26	0.797
孕酮（ng/ml）	0.36（0.22，0.61）	0.36（0.22，0.63）	0.30（0.19，0.38）	*Z*＝−1.16	0.247
AMH（ng/ml）	0.08（0.06，0.22）	0.08（0.06，0.17）	0.15（0.09，2.19）	*Z*＝−1.20	0.231
妇科干预治疗				*χ*^2^＝21.09	<0.001
激素替代治疗	137（52.1）	130（57.0）	7（20.0）		
传统草药干预	16（6.1）	14（6.1）	2（5.7）		
未行药物干预	110（41.8）	84（36.8）	26（74.3）		

**注** GnRH-a：促性腺激素释放激素激动剂；AA：再生障碍性贫血；AL：急性白血病；MDS：骨髓增生异常综合征；MPN：骨髓增殖性肿瘤；NHL：非霍奇金淋巴瘤；MAC：清髓性预处理方案；NMAC：非清髓性预处理方案；GVHD：移植物抗宿主病；AMH：抗米勒管激素；^a^具有性激素水平电子记录的患者共113例，其中移植后继发闭经组100例，移植后自然恢复月经组13例

二、生育力保护和保存

1. 移植前生育力保护和保存：56例（21.3％）患者采取生育保护和保存措施，其中51例患者移植前应用GnRH-a戈舍瑞林保护卵巢功能，5例患者接受卵母细胞冻存。

2. 移植后生育力保护和保存：临床医师和患者在移植后对生育力的关注程度较高，182例（69.2％）患者在移植后进行激素或妇科B超等生育功能评估；153（58.2％）例患者在妇科进行药物干预，其中137例患者采用HRT，16例患者选择传统草药干预。

三、生育力的影响因素分析

1. 患者移植后月经恢复的影响因素：263例患者中，228例（86.7％）患者继发闭经；35例（13.3％）自然恢复月经，其中26例月经规律、9例月经紊乱，中位恢复时间为2（1，4）年。137例患者接受以HRT为主的药物干预，134例（97.8％）患者用药后恢复月经，其中26例为激素依赖性月经；3例足量足周期用药后仍未恢复月经。

对移植后继发闭经和自然恢复月经组进行组间对比，得出预处理方案（*χ*^2^＝4.84，*P*＝0.028）与移植前生育力保护措施（*χ*^2^＝52.18，*P*<0.001）是月经恢复的影响因素。移植后自然恢复月经组接受GnRH-a治疗的患者比例高于移植后继发闭经组（65.7％对12.3％）且未行任何生育力保护的患者比例较低（28.6％对86.4％）。移植后继发闭经组接受MAC的患者比例高于移植后自然恢复月经组（87.3％对71.4％）。两组在移植年龄、疾病种类、移植前月经情况等方面差异均无统计学意义（均*P*>0.05，表1）。将*P*值小于或接近0.05的影响因素纳入Logistic回归模型中进行多因素分析（表2），去除混杂因素后结果显示，移植前使用GnRH-a是移植后患者月经恢复的独立影响因素（*OR*＝0.06，95％ *CI*：0.03～0.15，*P*<0.001）。

**表2:** 女性血液系统疾病患者移植后月经恢复的多因素分析

影响因素	多因素分析	
	*OR*（95％ *CI*）	*P*值
疾病种类		
AA	0（0～Inf）	0.997
AL	0.75（0.07～8.08）	0.815
MDS/MPN	0.77（0.05～11.90）	0.851
NHL	NA	NA
预处理方案		
MAC	0（0～Inf）	0.998
NMAC	NA	NA
移植类型		
同胞全相合	0（0～Inf）	0.988
单倍型	0（0～Inf）	0.987
无关全相合	NA	NA
移植前使用GnRH-a	0.06（0.03～0.15）	<0.001

**注** AA：再生障碍性贫血；AL：急性白血病；MDS：骨髓增生异常综合征；MPN：骨髓增殖性肿瘤；NHL：非霍奇金淋巴瘤；MAC：清髓性预处理方案；NMAC：非清髓性预处理方案；GnRH-a：促性腺激素释放激素激动剂；Inf：无穷大；NA：不适用［在多因素分析中，部分变量（如疾病种类中的NHL、预处理方案中的NMAC等）由于存在样本量过少或零单元格，导致模型无法计算出有效的*OR*（95％ *CI*），因此在表中标注为NA］

对药物干预后患者的月经恢复情况进行组间比较分析，结果显示月经恢复状况与移植年龄（*H*＝25.36, *P*<0.001）、预处理方案（*χ*^2^＝10.89, *P*＝0.004）、移植类型（*χ*^2^＝15.24, *P*＝0.003）、移植前使用GnRH-a（*χ*^2^＝65.18, *P*<0.001）及烷化剂（*χ*^2^＝6.35, *P*＝0.042）相关（表3）。未恢复月经组患者的移植中位年龄、接受MAC的患者比例高于自然恢复月经组和用药恢复月经组（均*P*<0.05）。自然恢复月经组（91.4％）和用药恢复月经组（78.4％）单倍型移植患者比例均高于未恢复月经组（69.1％）（*P*＝0.003）。移植前使用GnRH-a的51例（19.4％）患者中，86.3％的患者移植后月经恢复，其中自然恢复月经组与用药恢复月经组患者分别占45.1％和41.2％（*P*<0.001）。用药恢复月经组移植前使用烷化剂的患者比例（47.8％）最高（*P*＝0.042）。其余基线特征差异均无统计学意义（均*P*>0.05）。

**表3:** 接受异基因造血干细胞移植后的女性血液系统疾病患者药物干预治疗后月经恢复情况及比较［例（％）］

特征	自然恢复月经组（35例）	用药恢复月经组（134例）	未恢复月经组（94例）	统计量	*P*值
移植年龄［岁，*M*（*Q*_1_，*Q*_3_）］	19（15，25）	20（16，24）	24（19，27）	*H*＝25.36	<0.001
疾病种类				*χ*²＝12.82	0.073
AA	10（28.6）	21（15.7）	6（6.4）		
AL	21（60.0）	98（73.1）	75（79.8）		
MDS/MPN	3（8.6）	9（6.7）	9（9.6）		
NHL	1（2.9）	6（4.5）	4（4.3）		
移植前化疗疗程［个，*M*（*Q*_1_，*Q*_3_）］	2（0，3）	2（2，3）	3（2，3）	*H*＝3.51	0.172
移植前使用烷化剂	11（31.4）	64（47.8）	31（33.0）	*χ*²＝6.35	0.042
预处理方案				*χ*²＝10.89	0.004
MAC	25（71.4）	111（82.8）	88（93.6）		
NMAC	10（28.6）	23（17.2）	6（6.4）		
移植类型				*χ*²＝15.24	0.003
同胞全相合	3（8.6）	11（8.2）	21（22.3）		
单倍型	32（91.4）	105（78.4）	65（69.1）		
无关全相合	0（0）	18（13.4）	8（8.5）		
GVHD	26（74.3）	94（70.1）	69（73.4）	*χ*²＝0.41	0.816
移植前使用GnRH-a	23（65.7）	21（15.7）	7（7.4）	*χ*²＝65.18	<0.001
粒系重建时间［d，*M*（*Q*_1_，*Q*_3_）］	11（11，12）	11（11，12）	11（11，12）	*H*＝0.21	>0.05

**注** AA：再生障碍性贫血；AL：急性白血病；MDS：骨髓增生异常综合征；MPN：骨髓增殖性肿瘤；NHL：非霍奇金淋巴瘤；MAC：清髓性预处理方案；NMAC：非清髓性预处理方案；GVHD：移植物抗宿主病；GnRH-a：促性腺激素释放激素激动剂

2. 移植后激素水平与月经恢复：移植后对患者的性激素水平进行监测，共有113例患者有电子记录的激素水平，其中移植后继发闭经组100例、移植后自然恢复月经组13例。移植后继发闭经组患者的黄体生成素（LH）、卵泡刺激素（FSH）、AMH水平低于移植后自然恢复月经组，但差异均无统计学意义（均*P*>0.05，表1）。

3. 移植后实际生育情况：263例患者中仅有5例（1.9％）患者在移植后生育，1例患者为自然妊娠；4例患者使用辅助生殖技术，其中2例为移植前自体冻存卵母细胞复苏，1例为移植后自体卵母细胞复苏，1例为捐赠卵母细胞复苏。

## 讨论

allo-HSCT通过大剂量放化疗等预处理方案抑制免疫排斥，使移植物成功定植。这些治疗方式直接作用于卵巢，破坏卵泡储备和卵巢结构，引起早期卵巢卵泡耗竭，破坏卵巢间质微环境，特别是在卵泡发育的早期和中期[11]，从而导致女性生育力进行性下降、月经周期紊乱，最终可发展为POI与继发性不孕症。

根据移植前预处理方案的强度，将预处理分为MAC、NMAC。MAC预处理的患者发生POI的风险较高，且随着年龄增长以及TBI剂量增加而升高[12]–[13]。Sanders等[12]研究表明，144例接受含Cy和TBI的MAC方案的白血病患者中，仅9例恢复卵巢功能；而43例接受仅含Cy的NMAC方案的AA患者中，27例26岁以下患者的卵巢功能均恢复正常，而16例26岁以上患者中仅有5例恢复。联合TBI与不联合相比、分次照射与单次照射相比、未接受颅脑照射与接受相比，发生POI的比例更高[13]。本研究同样显示，86.7％的女性患者在allo-HSCT后出现继发闭经，而预处理方案中使用MAC是关键危险因素之一。本研究<18岁女性患者闭经比例较≥18岁患者低（83.4％对88.4％），与既往研究结果相似[14]。本研究仅纳入6例TBI患者，但均出现闭经（100％），而未使用TBI患者中86.4％继发闭经，与既往研究结果相似[15]–[16]。移植前行生育力保护是移植后生育力恢复的重要保护因素。GnRH-a可以抑制卵泡发育，促进性腺激素分泌，使卵泡处于静止状态，提高对性腺毒性的耐受性，显著降低POI发生率[17]。但是对于青春期前的女性，其下丘脑-垂体-性腺轴尚未建立，GnRH-a不能提高其耐受性[18]–[19]。对于GnRH-a是否能够降低性腺不良反应发生率尚且存在争议。一项前瞻性随机对照研究表明，淋巴瘤患者使用或不使用GnRH-a，随访1年后两组均有约20％患者发生POI[20]。而Blumenfeld等[21]发现在淋巴瘤患者中，移植前联合使用GnRH-a与预处理化疗可显著降低性腺不良反应发生率，将POI发生率从82％降至33％，但在白血病患者中并未观察到相同保护效应。本研究中共51例患者使用GnRH-a，其在移植后自然恢复月经组中的患者比例显著高于移植后继发闭经组，这可能与本研究纳入的患者较为年轻有关；提示GnRH-a对年轻女性白血病患者具有保护卵巢的作用。

移植后HRT是生育力保护的另一有效措施。《造血干细胞移植患者的妇产科管理专家共识》[22]中建议无禁忌证的POI患者均应接受HRT。临床上常见的HRT为雌孕激素序贯疗法。与合成激素相比，天然雌激素和孕激素的心血管和乳腺不良反应更小[23]。HRT能在缓解低雌激素症状的同时一定程度上预防心血管疾病和骨质疏松[24]。本研究中137例患者在allo-HSCT后接受HRT，其中，97.8％的患者在接受HRT后实现月经复潮。对于存在HRT禁忌证的患者，可以选用传统草药干预，主要包括黑升麻异丙醇萃取物、升麻乙醇萃取物，其作用机制可能与神经递质改变有关[25]。

卵母细胞冷冻是生育力保存的一线治疗方案，尤其适用于无配偶且治疗安排可延缓至少两周的成年未婚女性。冷冻与新鲜卵母细胞妊娠率无明显差异且未增加染色体异常、出生缺陷和发育缺陷的发生率[26]。需要注意的是，成熟卵母细胞冷冻需要2～4周促排卵时间，不能保存卵巢内分泌功能，且存在一定程度恶性细胞污染风险。卵巢组织冷冻技术（OTC）不仅能保护生育能力，且移植后卵巢可恢复内分泌功能和排卵。全球通过该技术出生200多名婴儿，冷冻的卵巢组织也可用于诱导患儿进入青春期[27]。本研究入组患者移植时间跨度较大，受限于疾病进展较快、生育力保存意识不足、技术受限等问题，仅5例患者成功进行卵母细胞冷冻，其中2例自体冻存卵母细胞成功复苏并生育。值得注意的是，本研究报道了1例确诊MDS的26岁女性患者，接受捐赠卵母细胞后成功生育，这与Zhang等[28]的报道类似，表明移植后POI的患者可通过受赠卵母细胞实现生育意愿，但应在社会伦理规范的前提下审慎实施。

本研究存在一定的局限性，本研究为单中心回顾性研究，移植时间跨度较大，不同时期临床医师与患者对生育力保护的认知存在差异；本研究仅113例患者包含性激素检验结果且检测时间点并不一致，缺乏影像学卵泡计数等反映卵巢储备结果；缺乏生育力保护和保存等干预措施对移植后生育力恢复和生育结局的前瞻性比较。未来本团队将综合患者年龄、疾病类型、拟采取治疗方案、卵巢功能评估等多参数构建卵巢损伤风险评估体系，开展多学科治疗模式，为患者制定个体化生育力保护和保存方案，通过系统性随访和多维度监测建立标准化管理路径。

综上所述，allo-HSCT前的放疗化疗和预处理都会造成不可逆性女性卵巢功能损伤。血液科医护团队需提高年轻血液系统疾病患者生育力保护和保存意识，包括应用GnRH-a保护卵巢、使用HRT缓解低雌激素症状并改善性腺功能，以及在充分评估患者治疗急迫性、机体耐受性等情况下选择最佳时机运用卵母细胞冷冻等辅助生殖技术保存患者生育力。
